# Diaspora-driven knowledge diplomacy: rethinking transnational education through the CIESDEMEX model in the Global South

**DOI:** 10.3389/frma.2026.1864987

**Published:** 2026-08-17

**Authors:** Daniel Arturo Romero León, Salvador Tapia Spinoso, Heidy Yelni Díaz Oviedo

**Affiliations:** 1 Instituto de Investigaciones y Estudios Superiores Económicos y Sociales (IIESES), Universidad Veracruzana, Xalapa, Mexico; 2 Facultad de Contaduría y Administración (FCA), Universidad Veracruzana, Xalapa, Mexico; 3 Facultad de Administración (FA), Universidad Veracruzana, Veracruz, Mexico

**Keywords:** brain circulation, CIESDEMEX, Global South, internationalization of higher education, knowledge diplomacy, scientific diaspora, transnational education

## Abstract

The migration of highly skilled professionals from the Global South has traditionally been framed as brain drain, emphasizing the loss of human capital and weakened national innovation systems; however, recent scholarship highlights the potential of scientific diasporas to enable knowledge circulation and transnational collaboration. This article aims to conceptualize how diaspora engagement, transnational education, and knowledge diplomacy can be integrated into a coherent framework for internationalization. Using a conceptual analysis approach, the study develops a multi-level model inspired by the CIESDEMEX initiative, examining how diaspora networks, higher education institutions, and policy actors interact to structure knowledge flows. The findings suggest that institutionalizing diaspora engagement and leveraging digital transnational education can transform fragmented exchanges into sustained systems of collaboration, while knowledge diplomacy provides the governance framework that aligns education, international cooperation, and development strategies. The article concludes that this integrated approach offers a pathway for Global South institutions to move beyond mobility-centered internationalization toward more inclusive, networked, and scalable models of knowledge circulation.

## 1 Introduction

Since the pioneering work of Johnson (1967), the migration of highly skilled professionals has largely been interpreted through the brain drain paradigm. The migration of scientists and highly skilled professionals from the Global South has historically been framed within the concept of brain drain, emphasizing the loss of human capital and the weakening of national scientific systems (Gibson and McKenzie, 2011). However, recent research has challenged this perspective by highlighting the role of scientific diasporas as active agents of knowledge circulation, innovation, and international collaboration (Meyer and Brown, 1999; Saxenian, 2007). In particular, growing empirical evidence shows that organized scientific diasporas can contribute not only to academic exchange but also to broader processes of science diplomacy and development in emerging economies (Echeverría-King et al., 2022).

Researcher mobility need not result in a one-way loss. It can generate brain circulation, in which knowledge, skills, and networks flow across borders in multiple directions (Tejada and Bolay, 2010). These dynamics enable diaspora communities to contribute to research collaboration, capacity building, and the strengthening of science, technology, and innovation (STI) ecosystems in their countries of origin (Kuznetsov and Sabel, 2006). Recent policy discussions also show that such contributions are more effective when supported by coordinated institutional frameworks and governance mechanisms that actively engage diaspora networks (Bonilla et al., 2023).

Parallel to the evolution of diaspora studies, the internationalization of higher education has expanded toward more complex forms of transnational collaboration. While traditional models were largely based on student mobility, recent approaches emphasize transnational education as a mechanism for facilitating knowledge exchange and expanding access across borders (Knight, 2016; de Wit and Altbach, 2021). This shift matters most where physical mobility is limited, making digital and hybrid modalities increasingly central to global academic engagement.

However, these processes often reproduce structural inequalities in global knowledge production, privileging institutions in the Global North (Marginson, 2016). In response, scholars have called for more inclusive models of internationalization that strengthen South-South cooperation and enhance the role of Global South institutions (Woldegiyorgis et al., 2018). Diaspora engagement can therefore help redistribute knowledge flows and expand participation in global academic networks.

The concept of knowledge diplomacy has gained relevance as a framework to understand how higher education and research contribute to international cooperation (Knight, 2022; Ruffini, 2020). Beyond traditional diplomatic channels, this perspective highlights the role of universities, academic networks, and diaspora communities as central actors in shaping global knowledge systems. Recent studies find that diaspora-driven initiatives can function as bridges between countries, facilitating collaboration and policy alignment across borders (Echeverría-King et al., 2022; Bonilla et al., 2023).

Within this emerging landscape, the Consorcio de Instituciones de Educación Superior para el Desarrollo Educativo de las personas Mexicanas en el Exterior (CIESDEMEX) represents an innovative institutional initiative promoted by the Mexican Government through the Secretaría de Relaciones Exteriores (SRE), the Instituto de los Mexicanos en el Exterior (IME), and the Secretaría de Educación Pública (SEP), together with a broad consortium of Mexican higher education institutions. CIESDEMEX does not focus exclusively on student mobility. It seeks to expand educational opportunities for Mexicans living abroad by coordinating universities, public institutions, and digital educational resources through a collaborative national framework.

Although CIESDEMEX was originally conceived as an educational initiative serving the broader Mexican diaspora, its institutional architecture also provides a valuable analytical lens for examining how higher education institutions, highly skilled diaspora communities, and governmental actors can jointly contribute to transnational knowledge circulation. CIESDEMEX thus illustrates an emerging model in which educational cooperation extends beyond academic provision to facilitate sustained international collaboration through institutional coordination.

This article does not treat CIESDEMEX as an empirical case study. It uses the initiative as a conceptual reference that helps integrate three bodies of literature that are frequently examined separately: scientific diasporas, transnational education, and knowledge diplomacy. Its value for this analysis lies not in demonstrating causal relationships, but in illustrating how these dimensions may be articulated within an institutional framework capable of supporting more coordinated forms of internationalization in the Global South.

Despite these advances, there is still limited research on how institutional platforms integrate transnational education and diaspora engagement to facilitate knowledge diplomacy. This gap is particularly evident in emerging initiatives in the Global South, such as CIESDEMEX, which remain underexplored in the academic literature (Orozco Aguirre, 2024). Addressing this gap, this article develops a conceptual analytical framework that integrates scientific diasporas, transnational education, and knowledge diplomacy through the institutional experience of CIESDEMEX as an illustrative conceptual lens. The article does not evaluate the initiative empirically; it examines how its institutional configuration can inform a broader understanding of diaspora-driven knowledge diplomacy and more networked approaches to internationalization in the Global South.

## 2 Conceptual analysis methodology

This study adopts a conceptual analysis approach to examine how scientific diasporas, transnational education, and knowledge diplomacy can be integrated into a coherent analytical framework for understanding emerging forms of internationalization in the Global South. Conceptual analysis does not test hypotheses with empirical data. It seeks to clarify, relate, and refine key concepts in order to generate new theoretical interpretations and analytical propositions (Jaakkola, 2020; MacInnis, 2011).

The article does not present CIESDEMEX as an empirical case study or as evidence for causal inference. Instead, it employs the initiative as an illustrative conceptual lens through which relationships among diaspora engagement, transnational education, and knowledge diplomacy can be interpreted. CIESDEMEX serves here as an institutional reference that facilitates theoretical reflection on how these dimensions may interact within a coordinated framework of internationalization.

The analytical process consisted of three complementary stages. First, a focused review of the literature on scientific diasporas, brain circulation, transnational education, knowledge diplomacy, and internationalization of higher education was undertaken. The review privileged peer-reviewed journal articles, scholarly books, and policy-oriented publications that have contributed to the conceptual development of these fields, while also incorporating recent studies addressing Global South perspectives and diaspora engagement. Particular attention was given to identifying areas of conceptual convergence and divergence across these bodies of literature.

Second, the concepts identified in the literature were comparatively examined to clarify their analytical boundaries and explore their complementarities. The analysis examined scientific diasporas, transnational education, and knowledge diplomacy jointly and focused on identifying their intersections and the institutional conditions under which they may jointly facilitate knowledge circulation across borders.

Third, these conceptual relationships were synthesized into a multi-level analytical framework organized around three interconnected dimensions: the micro level (diaspora actors and transnational networks), the meso level (higher education institutions and collaborative educational infrastructures), and the macro level (public policy, governance, and knowledge diplomacy). This heuristic framework is intended to organize existing theoretical insights and to provide a basis for future empirical research without claiming to validate causal relationships.

The analysis also draws on publicly available institutional documentation concerning CIESDEMEX to contextualize its organizational characteristics and educational objectives. These materials are used exclusively to describe the initiative and to support its interpretation as an emerging institutional experience, not as empirical evidence for testing the proposed framework.

As a conceptual contribution, this study has inherent limitations. The proposed framework has not yet been empirically validated through interviews, surveys, network analysis, or comparative case studies. The relationships discussed throughout the article should therefore be read as analytical propositions that require future empirical examination across different institutional and national contexts.

Generative AI tools were used exclusively for English-language editing and stylistic revision; they were not used to generate evidence, conduct analysis, or formulate the conceptual framework. Tool details and author verification procedures are reported in the Acknowledgments section.

## 3 Conceptual framework: integrating diasporas, transnational education, and knowledge diplomacy

The transformation of global knowledge systems has generated the need for analytical frameworks capable of capturing the complex interactions between mobility, education, and international cooperation. This section develops an integrated conceptual framework that brings together three key dimensions: scientific diasporas, transnational education, and knowledge diplomacy. While each of these fields has been studied independently, their intersection remains underexplored, particularly in relation to institutional mechanisms that enable knowledge circulation in the Global South.

### 3.1 Scientific diasporas and brain circulation

Scientific diasporas constitute a specific subset of highly skilled diasporas and should be distinguished from the broader notion of migrant diasporas. While migrant diasporas encompass diverse social, cultural, and economic communities living outside their countries of origin, scientific diasporas refer specifically to researchers, academics, scientists, and other highly qualified professionals who maintain sustained professional or research linkages across national borders (Meyer, 2001; Tejada and Bolay, 2010). The distinction matters in Mexico, where educational initiatives directed toward the broader diaspora coexist with policies specifically designed to engage highly skilled professionals and scientific communities abroad.

The concept of scientific diasporas has evolved significantly over the past decades, shifting from a focus on loss toward a recognition of their potential as strategic assets in global knowledge systems (Meyer et al., 1997). Early approaches, grounded in the brain drain paradigm, emphasized the negative consequences of skilled migration for developing countries, particularly in terms of human capital depletion (Gibson and McKenzie, 2011). However, more recent perspectives highlight the emergence of brain circulation as a more accurate representation of contemporary mobility patterns (Saxenian, 2007).

Contemporary understandings of brain circulation have also been enriched by studies examining skilled migration as part of global labor mobility and innovation systems (Docquier and Rapoport, 2012). Scientific diasporas can thus be understood as transnational networks of highly skilled individuals who maintain professional, academic, and social ties with their countries of origin (Meyer and Brown, 1999). These networks facilitate the multidirectional flow of knowledge, skills, and resources, enabling processes of co-production and innovation across borders (Tejada and Bolay, 2010). Diaspora communities actively contribute to research collaboration, mentoring, and institutional development, reinforcing the idea that knowledge is increasingly produced within distributed and networked systems (Wagner, 2008).

The effectiveness of diaspora engagement depends on the existence of institutional mechanisms that can channel these dispersed capabilities into structured forms of collaboration (Kuznetsov and Sabel, 2006). Without such mechanisms, diaspora contributions tend to remain fragmented and episodic. This highlights the need to move beyond individual-level analyses and toward institutional frameworks that enable scalable and sustained forms of knowledge circulation.

The existence of scientific diasporas alone does not guarantee effective knowledge circulation. As (Meyer and Brown 1999) have argued, institutional arrangements play a decisive role in transforming dispersed individual expertise into coordinated forms of academic cooperation. A study of scientific diasporas must therefore extend beyond individual mobility to the analysis of institutional mechanisms capable of sustaining long-term collaboration. This institutional perspective provides the conceptual bridge to transnational education discussed in the following section.

### 3.2 Transnational education as an operational infrastructure

In this article, the expression operational infrastructure refers to the institutional arrangements, educational platforms, collaborative mechanisms, and organizational processes that enable sustained academic interaction across borders. It does not imply that transnational education automatically generates collaboration; rather, it provides the institutional conditions under which collaborative activities may be organized, coordinated, and maintained over time. The evolution of diaspora studies, the internationalization of higher education has expanded beyond traditional mobility-based models toward more flexible and inclusive forms of transnational education (TNE). TNE encompasses a wide range of practices, including cross-border academic programs, digital learning environments, and collaborative institutional networks (Knight, 2016).

In this framework, transnational education may function as an operational infrastructure that facilitates the circulation of knowledge across geographical boundaries. Unlike conventional models that rely on the physical movement of students and faculty, TNE allows for the decentralization of educational processes, facilitating access to knowledge regardless of location (de Wit and Altbach, 2021). This function is especially important in the Global South, where structural barriers often limit participation in traditional internationalization schemes.

TNE also provides a platform through which diaspora communities can engage with academic institutions in their countries of origin. Through teaching, mentoring, and collaborative research conducted in virtual or hybrid formats, diaspora scientists can contribute to strengthening local capacities without the need for permanent return. Transnational education can then connect geographically dispersed actors, transforming mobility into sustained interaction.

TNE is not inherently equitable. Without deliberate design, it can reproduce existing asymmetries in global knowledge production, particularly when dominated by institutions from the Global North (Marginson, 2016). This underscores the importance of developing context-sensitive models of transnational education that prioritize reciprocity, inclusion, and local relevance.

### 3.3 Knowledge diplomacy as a coordinating framework

Following Knight (2022), knowledge diplomacy is not a synonym for internationalization or cross-border education. It refers to the strategic use of higher education, research, innovation, and knowledge exchange to strengthen relationships among countries through reciprocity, mutual benefit, and long-term collaboration. This distinction is particularly important because not every international educational activity necessarily constitutes knowledge diplomacy. The concept of knowledge diplomacy has emerged as a response to the growing recognition that higher education, research, and innovation are central to addressing global challenges. Unlike traditional forms of science diplomacy, which tend to be state-centric, knowledge diplomacy encompasses a broader range of actors, including universities, academic networks, and diaspora communities (Knight, 2022; Ruffini, 2020).

Contemporary science diplomacy literature increasingly recognizes the participation of universities, scientific communities, funding agencies, and transnational research networks. Instead, knowledge diplomacy offers a complementary perspective by incorporating higher education, lifelong learning, research, innovation, and educational cooperation within a broader framework of international relations.

Diaspora communities mediate between knowledge systems and facilitate the circulation of expertise across borders. Universities provide institutional hubs that enable these interactions, while governments provide the policy frameworks that support and regulate them. This multi-actor configuration reflects a shift toward more networked and decentralized forms of global governance (Echeverría-King et al., 2022).

Importantly, knowledge diplomacy provides the conceptual lens that allows us to understand how diaspora engagement and transnational education can be aligned within broader strategies of international cooperation. Its central concern is how coordination, institutional design, and policy integration can transform dispersed knowledge flows into structured and sustainable systems.

### 3.4 Toward an integrated conceptual framework

Bringing these dimensions together, this article proposes an integrated framework in which scientific diasporas, transnational education, and knowledge diplomacy are understood as interdependent components of a broader system of knowledge circulation. Scientific diasporas provide the human capital and transnational networks that enable the flow of knowledge; transnational education functions as the operational infrastructure that facilitates interaction and collaboration; and knowledge diplomacy acts as the coordinating framework that aligns actors, institutions, and policies.

Despite their complementarities, these dimensions are often treated separately in the literature, limiting our understanding of how they interact in practice. This fragmentation is particularly evident in the study of Global South initiatives, where institutional innovations remain under-theorized (Gacel-Ávila, 2018).

By integrating these elements, the proposed framework shifts the focus from isolated processes to systemic interactions. It allows for a more comprehensive understanding of how knowledge is produced, circulated, and institutionalized in contemporary global contexts. This synthesis provides the analytical foundation for conceptualizing CIESDEMEX not merely as a program, but as an institutional infrastructure of diaspora-driven knowledge diplomacy capable of enabling scalable, inclusive, and sustainable forms of international academic collaboration.

The proposed framework should therefore be interpreted as an analytical heuristic rather than a causal explanatory model. Its purpose is to organize conceptual relationships identified across different strands of literature and to provide a basis for future empirical research examining how institutional initiatives such as CIESDEMEX may facilitate knowledge circulation under different policy and organizational contexts. This study differs from existing approaches in three main ways. First, it integrates diaspora engagement, transnational education, and knowledge diplomacy into a single analytical framework, and examines their interaction. Second, it shifts the focus from mobility-centered internationalization toward network-based knowledge circulation. Third, it emphasizes the role of institutional infrastructures in structuring and sustaining these processes, particularly in the context of the Global South (Didou Aupetit, 2017; Gacel-Ávila, 2018).

## 4 The CIESDEMEX model as conceptual proposition

We use CIESDEMEX as a conceptual lens derived from an emerging institutional experience, not as an empirical case study. The model is used analytically to illustrate how diaspora engagement, transnational education, and knowledge diplomacy can be articulated into a structured system of knowledge circulation. This distinction is crucial for positioning the contribution of the article within a conceptual analysis framework.

CIESDEMEX is a collaborative initiative led by the Government of Mexico through the Ministry of Foreign Affairs (SRE), the Institute for Mexicans Abroad (IME), and the Ministry of Public Education (SEP), alongside a broad network of Mexican higher education institutions. It is not an independent educational provider; it functions as an inter-institutional consortium that coordinates universities, public agencies, and consular networks. Its primary objective is to expand educational opportunities for Mexicans residing abroad—particularly in the United States—by facilitating access to upper-secondary, undergraduate, graduate, continuing education, and lifelong learning programs to promote educational justice and social inclusion.

CIESDEMEX was formally launched in February 2022 through collaboration among the SRE, the IME, the SEP, and an initial group of Mexican higher education institutions [Instituto de los Mexicanos en el Exterior (IME), 2022a,b]. It was established as a non-profit, inter-institutional mechanism and not as a new university or government agency. Its General Operating Statutes, approved in 2022, define a collegial governance structure comprising a General Assembly, an Executive Commission, a presidency, a technical secretariat, and thematic working groups. The statutes also establish membership and decision-making procedures and frame participation as a form of shared institutional responsibility. In financial terms, the statutes contemplate resources generated with support from the IME and resources mobilized or generated through the consortium and its participating institutions (CIESDEMEX, 2022).

The consortium has expanded progressively since its creation. It comprised 72 Mexican public and private universities in 2024 and, following the incorporation of nine additional institutions during 2025, reported 81 member higher education institutions, two strategic partner associations, ANUIES (Asociación Nacional de Universidades e Instituciones de Educación Superior) and AMPEI (Asociación Mexicana para la Educación Internacional) and representation in 30 Mexican states in February 2026. At that time, its combined educational portfolio included 112 programs across continuing, upper-secondary, undergraduate, and graduate education [Instituto de los Mexicanos en el Exterior (IME), 2024; University of Guadalajara Foundation USA, 2026]. These figures describe the institutional reach of the consortium, but should not be interpreted as evidence of its educational or scientific impact, which has yet to be evaluated systematically.

Conceptually, this initiative reflects an emerging model of transnational educational governance where universities assume a collective role in strengthening institutional cooperation across national borders, effectively transforming the consortium into a tool for knowledge diplomacy. Under the leadership of the SRE, CIESDEMEX integrates higher education into the country's foreign policy framework. By collaborating closely with Mexican consulates and the Educational Orientation Windows (VOE), the initiative moves beyond traditional academic internationalization, actively connecting overseas communities with Mexico's broader cultural and educational advancement.

To address the geographic and employment constraints faced by migrants, the CIESDEMEX model is deeply rooted in flexible, virtual, and distance education, utilizing digital platforms and personalized assistance technologies to match academic offerings with the needs of the diaspora. The consortium also manages critical tasks regarding academic guidance, revalidation, and the accreditation of inter-institutional studies, which are vital for facilitating the educational reintegration of students and families who are part of return migration flows.

Structurally, this collaborative network coordinates a robust block of higher education institutions—including UNAM, the University of Guadalajara, Universidad Veracruzana, and UVEG, with the backing of ANUIES. Its primary mission is educational, although its architecture can also support scientific exchange. CIESDEMEX provides a unique institutional architecture within which highly skilled members of the diaspora—including academics, researchers, and professionals—can participate in teaching, mentoring, and research. Inspired by international models, this ecosystem leverages institutional diversity to explore how diaspora engagement, transnational education, and knowledge diplomacy can converge to drive social mobility.

Our framework treats CIESDEMEX as more than an internationalization initiative: it is a structured model that brings together diaspora engagement, transnational education, and coordinated institutional action. It organizes what would otherwise remain fragmented efforts—individual collaborations, isolated programs, or sporadic exchanges—into a more coherent and scalable system of knowledge circulation. This perspective becomes especially relevant in the Mexican case, where the size and diversity of the diaspora, along with its educational needs, have driven the creation of new institutional responses aimed at maintaining connections and expanding opportunities beyond national borders (Orozco Aguirre, 2024).

CIESDEMEX should also be understood within Mexico's broader ecosystem of diaspora engagement policies. The three principal mechanisms differ in their target populations and instruments. The IME's Red Global MX organizes highly qualified Mexican professionals abroad through regional and thematic networks to promote research collaboration, innovation, entrepreneurship, and knowledge transfer. SECIHTI's Cátedras de la Diáspora Mexicana is directed specifically at researchers and links them with public higher education institutions and research centers in Mexico through formal academic or research activities, recognition within the National System of Researchers, and mobility support. CIESDEMEX, by contrast, is primarily an educational consortium serving the wider Mexican population abroad by coordinating access to programs, guidance, credential recognition, and return pathways through Mexican higher education institutions. It therefore complements the scientific and professional orientation of Red Global MX and SECIHTI programs [Instituto de los Mexicanos en el Exterior (IME), n.d.; Secretaría de Ciencia, Humanidades, Tecnología e Innovación (SECIHTI), 2026].

### 4.1 Micro level: diaspora as distributed knowledge nodes

At the most immediate level, the model is sustained by the participation of the diaspora itself. Scientists, academics, and highly skilled professionals living abroad are not only carriers of expertise, but also active connectors between different academic and cultural environments. Through teaching, mentoring, collaborative research, and even informal exchanges, they contribute to building bridges between institutions and knowledge systems.

The contribution of these actors should not be interpreted as automatic. Scientific diaspora members differ substantially in terms of disciplinary backgrounds, institutional affiliations, professional trajectories, and willingness to engage with their countries of origin (Didou Aupetit, 2017). Institutional mechanisms are therefore needed to facilitate participation, reduce coordination costs, and transform dispersed expertise into sustained forms of collaboration.

In the Mexican context, this dimension acquires particular importance. The diaspora is not only large, but also diverse and increasingly qualified, which makes it a valuable resource for strengthening academic and scientific development (Orozco Aguirre, 2024). The model recognizes these individuals as part of a broader, distributed network of knowledge.

What CIESDEMEX does differently is to move beyond occasional or voluntary collaboration. It creates conditions for this participation to become more stable, organized, and meaningful over time. This arrangement can transform dispersed individual contributions into a collective capacity that can support innovation and knowledge production.

### 4.2 Meso level: institutional networks and coordination

Universities occupy a strategic position within this framework because they possess the organizational capacity to coordinate academic programs, recognize educational credentials, develop collaborative research agendas, and maintain long-term institutional partnerships. Here, transnational education denotes more than cross-border program delivery; it is an institutional mechanism through which knowledge circulation may be organized and sustained. At an intermediate level, these individual and networked interactions are translated into structured institutional collaboration. Universities play a central role here, acting as spaces where these connections take shape through academic programs, joint initiatives, and research projects.

One of the key mechanisms that makes this possible is transnational education, particularly through online and distance learning. These modalities allow members of the diaspora to remain academically connected without needing to relocate, making education more flexible and accessible (Orozco Aguirre, 2024). They also allow institutions in Mexico to extend their reach and integrate global perspectives into their academic offerings.

Equally important are the alliances that emerge between institutions. These partnerships, whether through joint programs, agreements, or collaborative platforms, help create a more stable environment for long-term cooperation. They also strengthen cultural and social ties, allowing diaspora communities to remain connected not only academically, but also in terms of identity and belonging (Orozco Aguirre, 2024).

Through these mechanisms, CIESDEMEX moves from isolated initiatives to a more organized system, where collaboration is not accidental but intentionally designed and sustained.

### 4.3 Macro level: knowledge diplomacy and governance

Knowledge diplomacy arises through coordinated interactions among ministries, universities, diaspora communities, and international partners. The diplomatic dimension therefore resides in the institutional capacity to create reciprocal educational relationships that simultaneously support international cooperation, national development, and the educational aspirations of Mexican communities abroad. At a broader level, we analyze the model through the concept of knowledge diplomacy. Here, different actors, as government institutions, universities, and diaspora communities, work together to align educational initiatives with broader national and international objectives.

In Mexico, institutions such as SRE, IME, and SEP play a key role in supporting and coordinating these efforts (Orozco Aguirre, 2024). Their involvement reflects a shift in how diplomacy is understood: not only as political negotiation, but also as a means of fostering educational opportunities and strengthening transnational ties.

Programs such as the Ventanillas de Orientación Educativa or the Semana Binacional de Educación illustrate how this coordination takes place in practice, connecting communities abroad with educational resources and opportunities (Orozco Aguirre, 2024). In this way, education becomes a bridge, not only between individuals and institutions, but also between countries.

Knowledge diplomacy provides the guiding logic that brings these different elements together. It allows educational initiatives to be aligned with broader goals of cooperation, development, and inclusion, giving coherence to what might otherwise be a set of disconnected actions.

### 4.4 CIESDEMEX as a multi-level infrastructure of knowledge circulation

When these three levels are considered together, CIESDEMEX can be seen as a multi-level system that connects people, institutions, and policies in a coordinated way. At the micro level, diaspora communities act as carriers and connectors of knowledge; at the meso level, universities and academic networks provide the structure for collaboration; and at the macro level, public institutions and diplomatic strategies create the conditions that make these interactions possible. This integration allows for a shift from fragmented exchanges to more sustained and scalable forms of collaboration. It also helps operationalize the idea of moving from brain drain to brain circulation, by creating channels through which knowledge can flow in multiple directions between diaspora communities and their countries of origin.

The model opens space for new forms of collaboration beyond traditional North-South dynamics. By leveraging diaspora networks, it becomes possible to connect institutions across different regions, fostering more horizontal and inclusive forms of knowledge exchange. CIESDEMEX is not only a program, but a way of organizing how knowledge can circulate in a more connected and equitable world. It offers a framework for understanding how internationalization can evolve—moving toward models that are more flexible, more inclusive, and better aligned with the realities of global mobility and digital interaction.

The framework is an analytical proposition, not an empirically validated model. The relationships described among diaspora engagement, transnational education, and knowledge diplomacy represent theoretically informed interpretations that require future empirical examination across different institutional settings. We use CIESDEMEX as an illustrative institutional experience that can inform broader discussions on internationalization in the Global South rather than as evidence of causal effectiveness.

## 5 Discussion: theoretical contributions

This study contributes to ongoing debates on internationalization, migration, and global knowledge systems by proposing an integrated conceptual framework that connects scientific diasporas, transnational education, and knowledge diplomacy. While these fields have often been examined separately, the CIESDEMEX model illustrates how their interaction can be institutionalized, offering a more comprehensive understanding of how knowledge circulates in the Global South. The article makes three theoretical contributions.

### 5.1 From brain drain to institutionalized brain circulation

First, the study extends the conceptual shift from brain drain to brain circulation by emphasizing its institutional dimension. Earlier approaches framed skilled migration primarily as a loss of human capital (Gibson and McKenzie, 2011), while more recent work has highlighted the role of diasporas in facilitating knowledge exchange and innovation across borders (Saxenian, 2007; Meyer and Brown, 1999). However, much of this literature still assumes that diaspora engagement occurs in dispersed and often informal ways (Tigau, 2015).

This article contributes by discussing how brain circulation can be actively structured through institutional mechanisms. In line with Kuznetsov and Sabel (2006), who stress the importance of policy frameworks in leveraging global talent, the CIESDEMEX model shows how transnational education, mentoring, and collaborative research can transform fragmented interactions into sustained systems of knowledge exchange. This perspective aligns with recent empirical evidence showing that organized scientific diasporas can significantly contribute to the strengthening of innovation systems and international collaboration when supported by structured initiatives (Echeverría-King et al., 2022).

By framing brain circulation as an institutionalized process, the study moves beyond descriptive interpretations and offers a more operational understanding of how mobility can strengthen science, technology, and innovation (STI) systems in the Global South.

### 5.2 Internationalization beyond mobility: a global south perspective

Second, the article contributes to the literature on the internationalization of higher education by offering a perspective rooted in the realities of the Global South. Traditional models of internationalization have often prioritized student mobility and global competition, reinforcing hierarchical relationships between institutions (de Wit and Altbach, 2021; Marginson, 2016). In contrast, more recent scholarship calls for more inclusive and context-sensitive approaches (Woldegiyorgis et al., 2018). The CIESDEMEX model responds to these calls by shifting the focus from mobility to connectivity. Through transnational education and diaspora engagement, it enables forms of collaboration that are not constrained by physical relocation, aligning with Knight's (2016) conceptualization of transnational education as a mechanism for expanding access and facilitating global knowledge exchange.

The model also shows the capacity of Global South institutions to actively shape internationalization processes. As suggested by recent work on diaspora engagement and policy-oriented collaboration, structured initiatives can redefine internationalization as a process of co-production instead of competition (Bonilla et al., 2023). In the Mexican context, strengthening educational linkages with diaspora populations further reinforces this shift toward more inclusive and network-based models (Orozco Aguirre, 2024).

### 5.3 Diaspora-driven knowledge diplomacy

Third, the study advances the concept of knowledge diplomacy by incorporating diaspora engagement as a central component. Existing approaches to science diplomacy have traditionally emphasized the role of states in fostering international cooperation (Ruffini, 2020), but recent work highlights the growing importance of non-state actors and networked governance structures (Ruffini, 2020; Echeverría-King et al., 2022).

Within this evolving landscape, scientific diasporas emerge as key intermediaries that connect knowledge systems and facilitate transnational collaboration. Empirical evidence shows that organized diaspora networks contribute not only to academic exchange but also to the articulation of science diplomacy strategies in emerging economies (Echeverría-King et al., 2022). This expands the scope of knowledge diplomacy beyond formal diplomatic channels, incorporating more flexible and decentralized forms of engagement.

The CIESDEMEX model exemplifies how these dynamics can be institutionalized. By integrating diaspora networks, universities, and government actors into a coordinated platform, it reflects a shift toward more collaborative and policy-oriented forms of knowledge diplomacy. This aligns with recent discussions emphasizing the role of institutional frameworks in structuring diaspora engagement as a tool for international cooperation and development (Bonilla et al., 2023).

### 5.4 Critical perspectives: governance, inequality, and institutional challenges

While the proposed framework highlights the potential of diaspora-driven knowledge diplomacy, it is equally important to recognize that knowledge circulation is neither automatic nor inherently equitable. Contemporary scholarship on internationalization and scientific diasporas emphasizes that transnational collaboration operates within asymmetrical global knowledge systems characterized by unequal access to resources, institutional prestige, funding opportunities, and research infrastructures (Marginson, 2016; Woldegiyorgis et al., 2018). CIESDEMEX and comparable initiatives cannot by themselves overcome these structural inequalities. Their contribution depends on governance arrangements capable of promoting reciprocity, inclusiveness, and long-term institutional commitment. Without such mechanisms, diaspora engagement may remain concentrated among already well-connected academics and institutions, and may reproduce existing disparities.

Scientific diasporas are themselves heterogeneous communities. Differences in disciplinary background, career stage, gender, migration trajectories, institutional affiliation, and access to international networks influence the forms and intensity of diaspora participation. Effective institutional strategies should therefore avoid assuming homogeneous patterns of engagement and instead recognize the diversity of actors, motivations, and capacities involved in transnational knowledge exchange. Governance therefore becomes a central dimension of diaspora-driven knowledge diplomacy. Beyond coordinating governmental agencies and universities, governance also involves defining priorities, allocating resources, establishing mechanisms for participation, and ensuring that educational cooperation generates reciprocal benefits for diaspora communities, higher education institutions, and public policy objectives. We interpret CIESDEMEX as an emerging institutional arrangement, not as a definitive solution to existing inequalities. Its long-term contribution will ultimately depend on its capacity to sustain inclusive, collaborative, and adaptable forms of international cooperation.

### 5.5 Toward an integrated understanding of knowledge circulation

The preceding discussion supports an integrated understanding of knowledge circulation in which migration, education, and diplomacy are analytically connected but remain institutionally distinct. The value of the proposed framework lies in identifying the conditions under which dispersed diaspora relationships may be translated into sustained forms of academic cooperation: accessible transnational educational arrangements, coordination among universities and public agencies, and governance mechanisms that promote reciprocity and inclusion. CIESDEMEX provides an illustrative institutional configuration through which these relationships can be examined, but does not demonstrate that such outcomes occur automatically. This synthesis therefore connects the theoretical contributions of the study with the policy and practical implications discussed in the following section, while leaving their effectiveness as a question for future empirical research.

## 6 Policy and practical implications

Understanding CIESDEMEX as a multi-level system of knowledge circulation offers several relevant insights for both policy design and institutional practice, particularly in the Global South. One of the main takeaways is the need to move beyond fragmented approaches toward more integrated policy ecosystems that connect education, science, innovation, and international cooperation. In many cases, these areas operate separately, which limits their overall impact. However, when they are aligned, diaspora engagement can become a strategic tool for strengthening collaboration and development. As recent studies suggest, coordinated policy frameworks allow diaspora initiatives to move from isolated efforts to more structured and impactful strategies (Bonilla et al., 2023). The CIESDEMEX model illustrates how this type of integration can create synergies between institutions and policy objectives.

A second key implication relates to the importance of institutionalizing diaspora engagement. While diaspora communities are widely recognized as valuable contributors to knowledge exchange, their participation often depends on personal initiative and remains irregular. This makes it difficult to sustain their contributions over time. By contrast, when engagement is supported through formal structures (such as academic programs, institutional agreements, and dedicated platforms) it becomes more stable and scalable. Research has shown that organized scientific diasporas can play a significant role in advancing science diplomacy and strengthening research systems when they are embedded within institutional frameworks (Echeverría-King et al., 2022). Institutionalization therefore means creating the conditions for long-term impact.

Another important implication concerns the growing role of digital and transnational education. The expansion of online and hybrid learning has made it easier to overcome geographic barriers, opening new possibilities for collaboration. For diaspora communities, this means they can participate in teaching, mentoring, and research activities without needing to return physically to their countries of origin. This type of engagement not only broadens access to education but also helps integrate global expertise into local contexts. As highlighted in recent policy discussions, digital education is becoming a key tool for scaling diaspora participation and facilitating knowledge exchange across borders (Bonilla et al., 2023). Sustaining this work requires investment in digital infrastructure, innovative teaching approaches, and regulatory frameworks that support cross-border education.

Finally, we argue for closer alignment among education, diplomacy, and development strategies. Higher education should not be seen only as a domestic policy issue, but also as a key component of international cooperation. When diaspora communities are effectively integrated into institutional and policy frameworks, they can act as bridges between countries, supporting collaboration and knowledge exchange. Evidence shows that organized diasporas can play a central role in science diplomacy, contributing to the strengthening of international networks and research capacities (Echeverría-King et al., 2022). Knowledge diplomacy provides a useful way of bringing together educational initiatives with broader foreign policy and development goals.

These implications call for a model of internationalization that does not rely mainly on student mobility. Institutions need more connected, flexible, and coordinated approaches that take advantage of diaspora networks and digital technologies. The CIESDEMEX model offers a clear example of how this can be done, showing that it is possible to build more inclusive, collaborative, and sustainable forms of engagement in global knowledge systems.

However, the implementation of such models is not without challenges. The institutionalization of diaspora engagement may face barriers related to bureaucratic fragmentation, uneven resource allocation, and digital inequalities that limit access to transnational education. Additionally, there is a risk that without careful design, these initiatives could reproduce existing asymmetries in knowledge production, privileging already well-connected actors. Addressing these challenges requires not only policy coordination but also a deliberate commitment to inclusivity and equitable participation.

## 7 Limitations and future research

While this study offers a conceptual contribution by proposing an integrated framework that connects scientific diasporas, transnational education, and knowledge diplomacy, we acknowledge several limitations. As a conceptual analysis, the article develops theory and does not provide empirical validation. The CIESDEMEX model is presented as a structured proposition based on existing literature and emerging practices, but its effectiveness and impact have not yet been systematically tested through quantitative or qualitative data. This means that some of the relationships identified—such as the link between institutionalized diaspora engagement and strengthened innovation systems—remain analytically grounded but require empirical confirmation.

A second limitation relates to the contextual nature of the case. Although CIESDEMEX provides a valuable example from the Global South, particularly within the Mexican context, its transferability to other regions may not be straightforward. Differences in institutional capacity, policy environments, and diaspora characteristics can influence how similar models operate in practice. As such, the framework should be understood as adaptable, not universally applicable, requiring contextual adjustments depending on regional and national conditions.

Additionally, the analysis places particular emphasis on institutional coordination and policy alignment, which may overlook some of the challenges associated with implementation. Issues such as bureaucratic fragmentation, limited funding, digital divides, and unequal access to educational opportunities can significantly affect the viability of initiatives like CIESDEMEX. Diaspora engagement is also heterogeneous; levels of participation, motivation, and connectivity vary widely across communities, which may influence the outcomes of such strategies.

These limitations open several avenues for future research. One important direction is the need for empirical studies that assess the impact of diaspora-driven initiatives on academic collaboration, knowledge production, and innovation outcomes. Comparative studies across countries or regions could also help identify the conditions under which models like CIESDEMEX are most effective, contributing to a more nuanced understanding of how institutional and policy environments shape knowledge circulation.

Future research could also explore the role of digital technologies in greater depth, particularly in relation to the quality and sustainability of transnational education. While digital platforms expand access, questions remain regarding learning outcomes, student engagement, and long-term institutional integration. Similarly, there is scope to examine how different forms of governance, centralized vs. network-based, for example, affect the coordination of diaspora engagement and knowledge diplomacy initiatives.

Another promising area of inquiry concerns the broader implications of diaspora engagement for equity and inclusion. While these models have the potential to democratize access to knowledge, they may also reproduce existing inequalities if not carefully designed. Understanding how to ensure more inclusive participation, across regions, institutions, and social groups, remains a key challenge for both researchers and policymakers.

In sum, this study provides a conceptual starting point for rethinking internationalization through the lens of diaspora-driven knowledge diplomacy. Future research will be essential to test, refine, and expand this framework, contributing to a deeper understanding of how knowledge can circulate in more inclusive, coordinated, and sustainable ways across global contexts.

## 8 Conclusion

This article has argued that the growing relevance of scientific diasporas, combined with the expansion of transnational education and the emergence of knowledge diplomacy, is reshaping how knowledge circulates in a globalized world. The study brought these processes together in an integrated framework, using the CIESDEMEX model as a conceptual reference point. This approach recasts internationalization not only as a set of activities, but as a coordinated system of actors, institutions, and policies.

One of the central conclusions is that diaspora engagement should no longer be seen as an auxiliary or voluntary dimension of internationalization. When properly structured, it can become a strategic component of national and institutional development. The case of CIESDEMEX illustrates how the articulation of diaspora networks, higher education institutions, and public policy can transform dispersed knowledge flows into more stable and meaningful forms of collaboration. This shift is particularly significant for countries in the Global South, where strengthening science, technology, and innovation systems remains a key priority.

The analysis also questions mobility-centered models of internationalization. While student mobility continues to play an important role, it is no longer sufficient to address the complexities of contemporary global knowledge systems. Digital and transnational education, along with network-based forms of collaboration, offer new possibilities for expanding access, fostering inclusion, and maintaining sustained engagement across borders. Internationalization, on this account, depends less on movement than on durable connections.

Beyond its theoretical contribution, this article invites a broader rethinking of internationalization as a strategic and coordinated process. In a context marked by increasing global interdependence and persistent inequalities, diaspora-driven knowledge diplomacy offers a pathway toward more inclusive and sustainable forms of engagement. By positioning Global South institutions as active agents in shaping knowledge circulation, this approach opens new possibilities for redefining the architecture of global knowledge systems in the 21st century.
